# Thermal Intravesical Chemotherapy Reduce Recurrence Rate for Non-muscle Invasive Bladder Cancer Patients: A Meta-Analysis

**DOI:** 10.3389/fonc.2020.00029

**Published:** 2020-02-05

**Authors:** Kang Liu, Jun Zhu, Yu-Xuan Song, Xiao Wang, Ke-Chong Zhou, Yi Lu, Xiao-Qiang Liu

**Affiliations:** Department of Urology, Tianjin Medical University General Hospital, Tianjin Medical University, Tianjin, China

**Keywords:** thermal intravesical chemotherapy, normal temperature intravesical chemotherapy, hyperthermic intravesical chemotherapy, external thermal field thermotherapy, non-muscle invasive bladder cancer, meta-analysis

## Abstract

**Background:** Non-muscle invasive bladder cancer accounts for nearly 80% of newly diagnosed bladder cancer cases, which often recur and progress. This meta-analysis was evaluated by the adverse events and recurrence rate of thermal intravesical chemotherapy vs. normal temperature intravesical chemotherapy in the treatment of non-muscle invasive bladder cancer.

**Methods:** A systematic review and cumulative analysis of studies reporting adverse events and recurrence rate of thermal intravesical chemotherapy vs. normal temperature intravesical chemotherapy was performed through a comprehensive search of Pubmed, Embase, Cochranelibrary.com, CNKI, Wanfang Med Online database and VIP database. All analyses were performed using the Revman manager 5.

**Result:** Twelve studies (11 randomized controlled trials and 1 retrospective study) including 888 patients, 445 in the thermal intravesical chemotherapy group, and 443 in the normal temperature intravesical chemotherapy group, met the eligibility criteria. Patients in the thermal intravesical chemotherapy group had a lower risk of disease recurrence than those who had normal temperature intravesical chemotherapy (24 months follow-up group: RR = 0.30, 95% CI: 0.21–0.43, *P* < 0.00001, *I*^2^ = 0%; 36 months follow-up group: RR = 0.27, 95% CI: 0.14–0.54, *P* = 0.0002, *I*^2^ = 0%) while no significant difference in adverse events rate (RR = 0.89, 95% CI = 0.53–1.52; *P* = 0.67, *I*^2^ = 78%).

**Conclusions:** When compared with normal temperature intravesical chemotherapy, thermal intravesical chemotherapy can reduce the recurrence rate without increasing incidence of adverse events in patients with non-muscle invasive bladder cancer.

## Introduction

Bladder cancer is one of the most common tumors in the urology system. In terms of its morbidity, it ranks the fourth among men and eleventh among women in all kinds of tumor, respectively (1). Last year, bladder cancer contributed more than 500,000 new cases and 190,000 deaths in 185 countries (2). Non-muscle invasive bladder cancer (NMIBC) makes up nearly 80% of newly diagnosed bladder carcinoma cases, of which Tis accounts for 10%, T1 for 30%, and Ta for 60% (3). Although the prognosis of patients with NMIBC have made great progress over the past decades, the recurrence and progression rate is still high. More than 60% of NMIBC patients will recur and more than 20% will progress into higher stages (4). Therefore, the economic burden created by intensive treatment and surveillance of NMIBC is very heavy for both individuals and governments.

Transurethral resection of bladder tumor (TURBT) is the most important diagnostic method and also the main treatment of NMIBC, but it could not prevent the recurrence and progression (5). Thus, adjuvant intravesical therapy came into being. It consists of bladder infusion chemotherapy and immunotherapy (6). One of the most effective intravesical therapy is bacillus Calmette-Guerin (BCG), which requires special care for its bio toxicity (7). And many other drugs, including mitomycin C (MMC), pirarubicin (THP), gemcitabine (GEM), hydroxycamptothecine (HCPT), etc. have been applied to intravesical chemotherapy (8). Despite all these efforts, the recurrence rate remains at 30% (9). In a word, preventing recurrence of NMIBC after TURBT still remains a challenge (10).

In recent years, thermal therapy has received increasing attention as a treatment for malignant tumors (11). High temperatures may enhance drug function by encouraging tumor cells to absorb more chemotherapeutic agents, redistributing their intracellular concentrations, altering metabolic patterns and/or inhibiting repair of DNA damage (12). Since NMIBC is prone to recurrence, thermal intravesical chemotherapy has been developed to improve the effectiveness of the treatment (13). It seems that thermal intravesical chemotherapy is good for patients with NMIBC (14). This meta-analysis is aimed to discuss whether thermal intravesical chemotherapy is associated with better efficacy with less or at least the same adverse events than normal temperature intravesical chemotherapy.

## Methods

### Eligibility Criteria

Studies were suitable for inclusion if they meet the following criteria: (1) participants: NMIBC patients receiving TURBT; (2) intervention: thermal intravesical chemotherapy; (3) control: normal temperature intravesical chemotherapy; (4) containing both of the following outcomes: recurrence rate and adverse event; (5) study design: randomized controlled trials (RCTs) or retrospective studies. The adverse event is as follow: cystitis, bladder irritation, hematuria, urinary pain, lower urinary tract symptoms, urinary tract infection, anorexia, anxiety, insomnia, rash, lower abdomen skin redness, fatigue, myelosuppression, influenza-like symptoms, and abnormal blood biochemical indexes. Exclusion criteria are as follows: (1) thermal intravesical chemotherapy was discontinued during the treatment schedule; (2) data cannot be obtained even after contacting the author; (3) duplicated publications. When multiple studies were delivered by the same researcher based on similar patients, only the most comprehensive or largest one was included.

### Study Search and Selection

Eligible studies focusing on the topic were identified through searching Pubmed, Embase, Cochranelibrary.com, CNKI, Wanfang Med Online database and VIP database. The search strategy is given in Appendix I. We also browsed reference lists of systematic reviews on this topic to find any other qualified articles. All searches without language limits but limited to studies on humans.

Two independent reviewers (LK and ZJ) examined the titles and abstracts according to eligibility criteria mentioned before. Studies underwent full-text examination after removing duplicated, irrelevant, review, case report, letter, editorial and non-comparative design studies. Divergences were resolved by discussion with another reviewer (S-YX).

### Quality Assessment and Data Extraction

The quality of all included RCTs was assessed using the “risk of bias” tool recommended by the Cochrane Collaboration. It consists of the following domains: random sequence generation, allocation concealment, blinding of participants and personnel, blinding of outcome assessment, incomplete outcome data, selective reporting and other bias. The Newcastle-ottawa quality scale was used to assess the quality of retrospective studies. Two reviewers (LK and ZJ) independently evaluated the quality of studies in these domains.

Data extraction was also executed by two reviewers (LK and ZJ) independently. The following information was extracted: first author's name, year of publication, study period, study design, sample size, clinical protocols, and number of patients who completed the study, age of participants, median follow-up, treatment schedule, and relevant data on outcomes. Disagreements were discussed and consensus was finally achieved.

### Statistical Analysis

Relative risks (RR) with 95%CIs for the adverse events rate were calculated to evaluate the safety of thermal intravesical chemotherapy, as well as for the recurrence rate of different follow-up groups to assess the effectiveness. Chi-squared tests were used to detect heterogeneity between studies included in this meta-analysis. Considering that the statistical power of the heterogeneity test is generally low, a *P*-value of 0.10 was set as the significance threshold for the heterogeneity. The heterogeneity was considered significant if *P* ≤ 0.1. We used *I*-squared (*I*^2^) statistic to indicate the proportion of variation between the studies due to heterogeneity. The larger the *I*^2^ value represented, the higher the heterogeneity was. And *I*^2^ > 50% suggested substantial heterogeneity among the studies. Fixed effect model was adopted when no significant heterogeneity was detected (*P* > 0.1 and *I*^2^ < 50%), otherwise, random effect model would be used. We did subgroup analyses between two treatment regimens according to the clinical protocols of study (hyperthermic intravesical chemotherapy vs. external thermal field thermotherapy), chemotherapeutic agent used in chemotherapy (MMC, THP, GEM, HCPT). All statistical analyses were performed using Revman software (version 5.3, The Cochrane Collaboration).

## Results

### Study Selection

**Figure 1:**
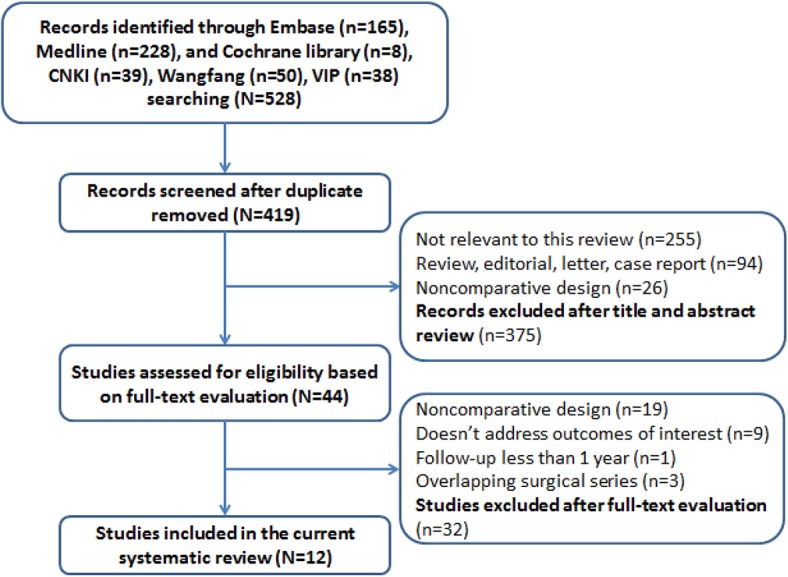
Flow diagram of study selection in the meta-analysis.

Five hundred and twenty-eight studies were identified from the aforementioned databases. One hundred and nine duplicated studies were first removed. Four hundred and nineteen studies were under screening titles and abstracts, among which 14 potentially relevant studies were obtained, and full texts were carefully checked for eligibility examination. Finally, 12 studies with a total of 888 participants were included in the meta-analysis. The process of study selection is shown in Figure 1.

### Study Characteristics

The characteristics of the included studies are summarized in Table 1. These studies were published between 2003 and 2019, 11 studies with an RCT design and one, a retrospective study. A total of 888 participants were enrolled, with a median size of 74 (ranging from 40 to 150). All of the studies had enrolled patients with NMIBC. The median duration of follow-up across the studies was 24 months. Treatment schedules varied between these studies.

**Table 1:** Characteristics of the studies included in the meta-analysis.

**Reference**	**Study period**	**Study design**	**Samplesize**	**Clinical protocals**	**Number of patients**	**Age (yrs)**	**Median follow-up (month)**	**Tumor stage**	**Pathological grade**	**Significantly differ** **between groups**	**Previous intravesical treatment**	**Treatmentdevice**	**Treatmentschedule**	**Dose**	**Temperature (°C)**	**Duration (min)**	**Severity of AE**	**AE**
Zhao et al. (15)	2011–2016	Single-center RCT	150	BCG	48	65.0 ± 7.1	24	NR	NR	No	NR	LR-2005 external thermal field treatment system (Guangzhou Laiwei Medical Devices Co., Ltd. Guangzhou, China)	**BCG:** 2 weeks after TURBT, BCG (150 mg) once a week for 6 weeks, then perfusion enhancement was performed at 3, 6, and 12 months, respectively. **ETFT-MMC:** 1 week after TURBT, MMC (30 mg) perfusion associated with ETFT once a week for 8 weeks, then once a month for 12 months. **MMC:** 1 week after TURBT, MMC (30 mg) once a week for 8 weeks, then once a month for 12 months.	**–**	NT	NR	Mild	Two influenza-like symptoms
				ETFT-MMC	49	67.0 ± 5.2	24							30 mg/30 ml	41~43	60		Three lower abdomen skin redness
				MMC	48	66.0 ± 5.4	24							30 mg/30 ml	NT	NR		0
Colombo et al. (16)	1994–1999	Multicentre RCT	83	ETFT-MMC	39	≤95:>65 = 25:17	24.0	Ta:T1:CIS = 15:26;1	G1:G2:G3 = 4:27:11	No	Having not received either local or systemic chemotherapy or radiotherapy during the last 3 months.	Synergo101–1 (Medical Enterprises, Amsterdam, the Netherlands)	**ETFT-MMC:** 20–40 days after TURBT, an induction cycle of 8 weekly sessions and a subsequent maintenance regimen of 4 monthly sessions. **MMC:** 20–40 days after TURBT, an induction cycle of 8 weekly sessions and a subsequent maintenance regimen of 4 monthly sessions.	20 mg/50 ml	40~44	40~60	Various	Thirty-four have different side effects of different severity
				MMC	36	≤65:>65 = 16:25	24.0	Ta:T1 = 17:24	G1:G2:G3 = 1:33:7					20 mg/50 ml	NT	60		Twenty-one have different side effects of different severity
Gao et al. (17)	2009–2012	Single-center RCT	64	HIVEC-MMC	32	54.9 ± 8.1	36.0	T1	G1:G2 = 16:16	No	NR	NR	**HIVEC-MMC:** 1 week after TURBT, MMC (30mg) once a week for 6 weeks, then every 2 weeks for six rounds. **MMC:** 1week after TURBT, MMC (30mg) once a week for 6 weeks, then every 2 weeks for six rounds.	30 mg/500 ml	42~43	120	Mild	Six bladder irritation
				MMC	32	56.5 ± 5.6	36.0		G1:G2 = 14:18					30 mg/500 ml	NT	NR		Seven bladder irritation
Guo et al. (18)	2013–2015	Single-center RCT	84	HIVEC-GEM	42	77.0 ± 6.0	24.0	Tis:TaG1/G2:G3:T1 = 3:20:11:8		No	NR	NR	**HIVEC-GEM:** Within 6 h after TURBT, GEM (1,000 mg) hyperthermic perfusion, then once a week for 8 weeks, after that every months for 1 year.**GEM:** Within 6 h after TURBT, GEM (1,000 mg) normal temperature perfusion, then once a week for 8 weeks, after that every months for 1 year.	1,000 mg/500 ml	42~44	120	Mild	Two hematuria and three urinary pain and four cystitis and three anorexia and three anxiety and two insomnia and one rash
				GEM	42	76.0 ± 7.0	24.0	Tis:TaG1/G2:G3:T1 = 4:20:12:6						NR	NT	NR		Eight hematuria and 10 urinary pain and 11 cystitis and 4 anorexia and 3 anxiety and 3 insomnia and 1 rash
Guo et al. (19)	2014–2016	Single-center RCT	74	HIVEC-THP	38	75.9 ± 5.7	24.0	Ta:T1 = 20:18	G1:G2 = 23:15	No	NR	NR	**HIVEC-THP:** Within 6 h after TURBT, THP (40 mg) hyperthermic perfusion, then once a week for 8 weeks, after that every months for 1 year. **THP:** 1 week after TURBT, THP (40 mg) normal temperature perfusion, then once a week for 8 weeks, after that every months for 1 year.	40 mg/45 ml	42~44	NR	Mild	Repeated calculation
				THP	36	75.0 ± 5.8	24.0	Ta:T1 = 19:17	G1:G2 = 24:12					40 mg/45 ml	NT	NR		
Li et al. (20)	2011–2014	Single-center RCT	90	HIVEC-MMC	45	58.4 ± 10.2	NR	T1	G1:G2 = 16:29	No	NR	BR-TRG-I type high-precision hyperthermic intraperitoneal perfusion treatment system	**HIVEC-MMC:** 3 days after TURBT, MMC (80 mg) hyperthermic perfusion, three times a day for four rounds. **MMC:** Within 24 h after TURBT, MMC normal temperature perfusion, then every 3 days for four rounds.	80 mg/600 ml	43	45	Mild	Ten bladder irritation
				MMC	45	60.4 ± 10.2	NR		G1:G2 = 14:31					NR	NT	45		Seven bladder irritation and one myelosuppression and one abnormal blood biochemical indexes
Liu et al. (21)	2009–2011	Single-center RCT	56	ETFT-THP	27	48.0~84.0	24.0	NR	low grade: high grade = 34:22	NR	No	ZD-2001 external thermal field treatment system	**ETFT-THP:** after TURBT, THP (40 mg) perfusion associated with ETFT once a week for 6 weeks, then THP (40 mg) only once every 2 weeks for 6 rounds, after that every months for six months. **THP:** after TURBT, THP (40 mg) once a week for 6 weeks, then once every 2 weeks for six rounds, after that every months for 6 months.	40 mg/40 ml	41~43	60	Mild	Five LUTS and two abnormal blood biochemical indexes
				THP	29		24.0							40 mg/40 ml	NT	30		13 LUTS and one abnormal blood biochemical indexes
Liu et al. (22)	2011–2014	Single-center RCT	40	HIVEC-MMC	20	51.5 ± 20.2	36.0	T1	G1:G2 = 18:22	No	NR	NR	**HIVEC-MMC:** 1 week after TURBT, MMC (30 mg) hyperthermic perfusion once a week for 6 weeks, then once every 2 weeks for six rounds. **MMC:** 1 week after TURBT, MMC (30 mg) once a week for 6 weeks, then once every 2 weeks for six rounds.	30 mg/500 ml	42~43	120	Mild	Four hematuria and 10 bladder irritation
				MMC	20		36.0							30 mg/500 ml	NT	NR		Two hematuria and eight bladder irritation
Peng et al. (23)	2010–2012	Single-center RCT	86	HIVEC-THP	44	42.0~68.0	22.3	Ta:T1 = 24:20	G1:G2 = 23:21	No	No	BR-TRG-I type high-precision hyperthermic intraperitoneal perfusion treatment system	**HIVEC-THP:** 1 week after TURBT, THP (40 mg) hyperthermia perfusion once a week for 8 weeks, after that every months for 8 months. **THP:** 1 week after TURBT, THP (40 mg) once a week for 8 weeks, after that every months for 8 months.	40 mg/600 ml	45	60	Mild	One gross hematuria
				THP	42		22.3	Ta:T1 = 23:19	G1:G2 = 10:32					40 mg/50 ml	NT	60		0
Su et al. (24)	2012–2014	Single-center RCT	76	HIVEC-MMC	38	50.2 ± 7.3	36.0	NR	NR	No	NR	NR	**HIVEC-MMC:** After TURBT, MMC (30 mg) hyperthermic perfusion once a week for 6 weeks, then twice a month for six rounds. **MMC:** After TURBT, MMC (30 mg) once a week for 6 weeks, then twice a month for six rounds.	30 mg/300 ml	45	120	Mild	One bladder irritation and one urinary tract infection
				MMC	38	50.5 ± 7.6	36.0							30 mg/500 ml	NT	NR		Four bladder irritation and four urinary tract infection and three fatigue
Zhao et al. (25)	2009–2014	Single-center RCT	83	ETFT-HCPT	39	65.0 ± 7.1	24.0	Ta:T1 = 15:27	G1:G2:G3 = 4:27:11	No	NR	LR-2005 external thermal field treatment system (Guangzhou Laiwei Medical Devices Co., Ltd. Guangzhou, China)	**ETFT-HCPT:** Within 24 h after TURBT, HCPT (20 mg) perfusion associated with ETFT, then once a week for 8 weeks, after that once a month for 6 months. **HCPT:** Within 24 h after TURBT, HCPT (20 mg) perfusion only, then once a week for 8 weeks, after that once a month for 6 months.	20 mg/40 ml	41~43	60	Mild	Repeated calculation
				HCPT	37	67.0 ± 5.2	24.0	Ta:T1 = 17:24	G1:G2:G3 = 1:33:7					20 mg/40 ml	NT	60		
Wang et al. (26)	2010–2015	Single-center Retrospective	74	HIVEC-THP	37	62.2 ± 7.4	24.0	Ta:T1 = 19:18	low grade: high grade = 27:10	No	No	BR-TRG-I type high-precision hyperthermic intraperitoneal perfusion treatment system	**HIVEC-THP:** Within 24 h after TURBT, THP (30 mg) hyperthermic perfusion, then once a week for 8 weeks, after that once a month. **THP:** Within 24 h after TURBT, THP (30 mg) perfusion, then once a week for 8 weeks, after that once a month.	30 mg/1,500 ml	43	60	Mild	Five bladder irritation
				THP	37	61.5 ± 7.2	24.0	Ta:T1 = 21:16	low grade: high grade = 29:8					30 mg/50 ml	NT	60		Two bladder irritation

*RCT, randomized controlled trial; ETFT, external thermal field thermotherapy; HIVEC, hyperthermic intravesical chemotherapy; MMC, mitomycin C; THP, pirarubicin; GEM, gemcitabine; HCPT, hydroxycamptothecine; NT, normal temperature; NR, not report*.

### Quality Assessment

**Figure 2:**
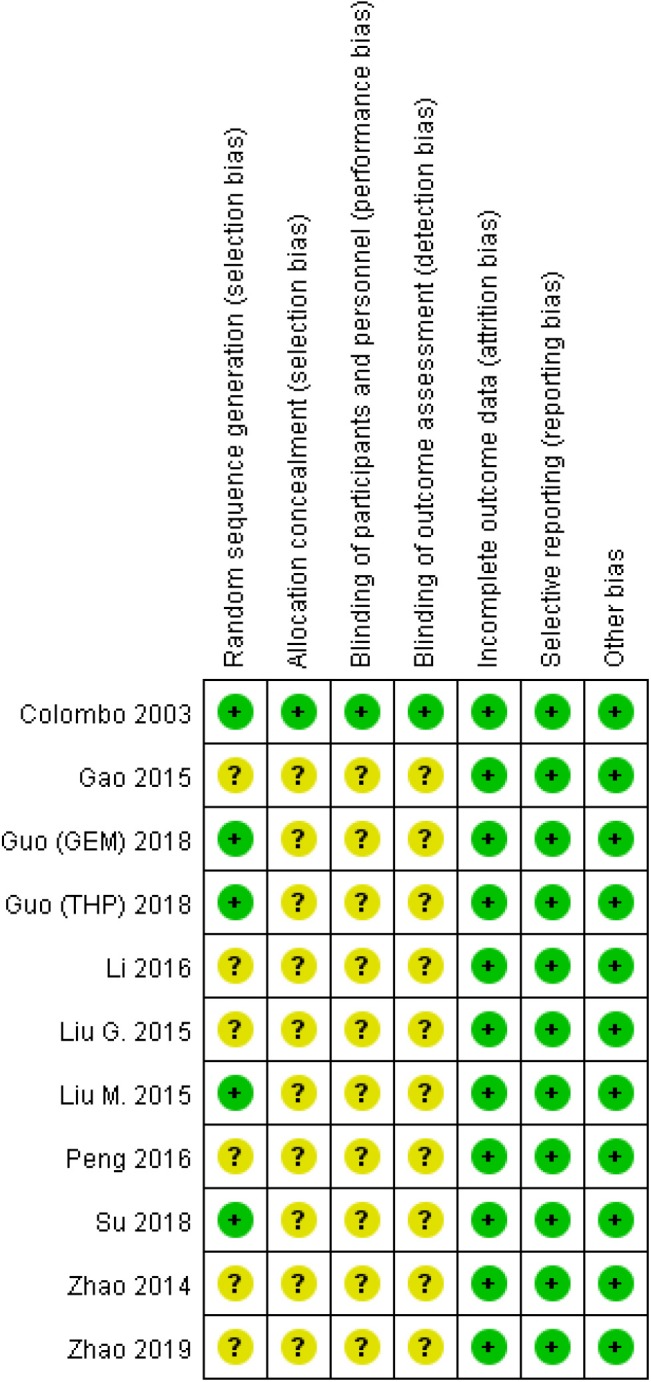
Risk of bias assessment of included studies.

The “risk of bias” tool recommended by the Cochrane Collaboration was adopted to assess the quality of included RCTs (Figure 2). Five studies (16, 18, 19, 22, 24) described how random sequence was generated, and all RCTs except Colombo et al. didn't describe the allocation concealment and Blind method. No incomplete or selective outcome data was reported. Quality assessments of cohort studies were conducted according to the Newcastle-Ottawa Scale (NOS), which was developed to assess bias risk including three domains with eight items (Table 2).

**Table 2:** Newcastle-ottawa quality scale.

	**Wang et al**.
Is the case definition adequate?	1
Representativeness of the cases	1
Selection of Controls	1
Definition of Controls	1
Comparability of cases and controls on the basis of the design or analysis	1
Ascertainment of exposure	1
Same method of ascertainment for cases and controls	1
Non-response rate	1
Total	8

### Recurrence Rate

**Figure 3:**
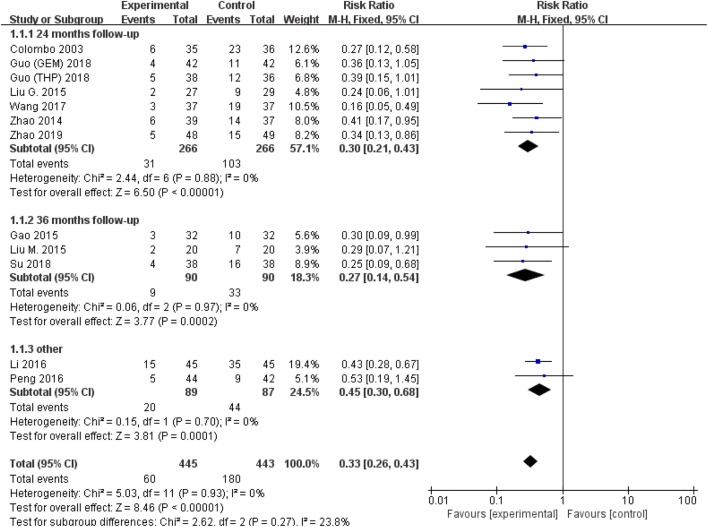
Forest plot of recurrence rate comparing thermal intravesical chemotherapy with normal temperature intravesical chemotherapy in different follow-up groups.

**Figure 4:**
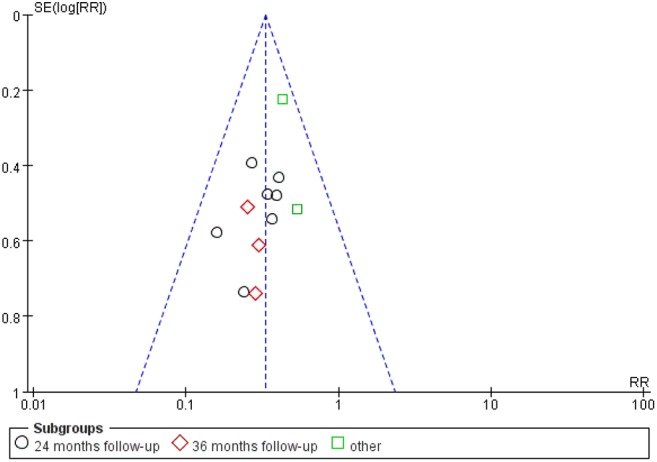
Funnel plot of included studies.

**Figure 5:**
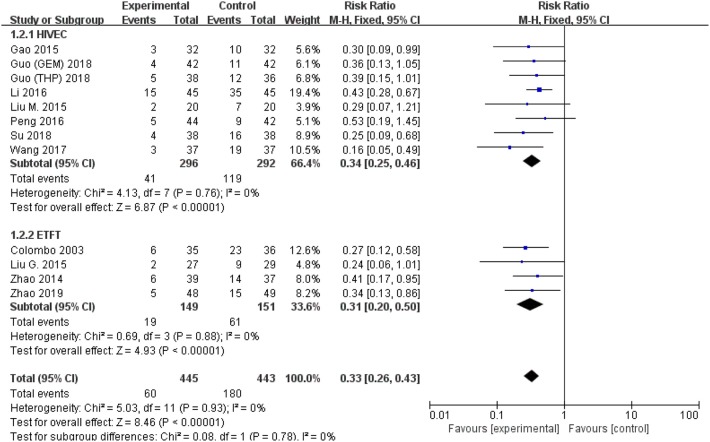
Comparison on recurrence rate between thermal intravesical chemotherapy and normal temperature intravesical chemotherapy after subgroup analysis stratified by different approach used in thermal therapy.

**Figure 6:**
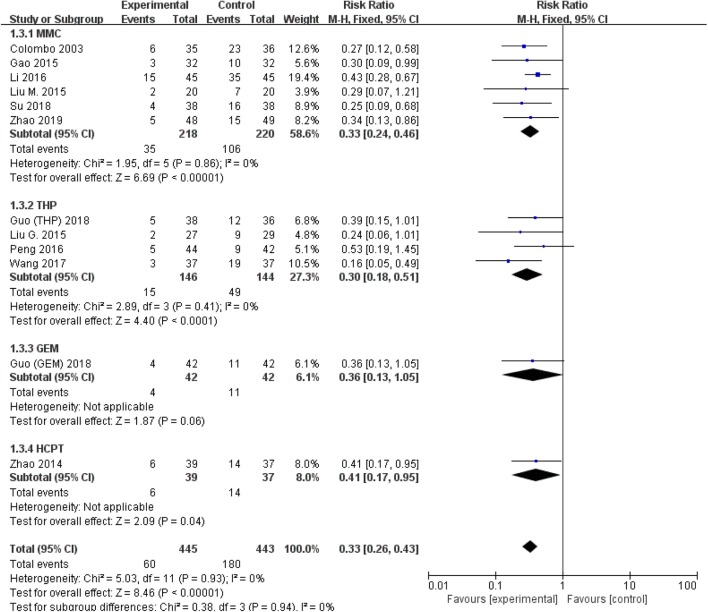
Comparison on recurrence rate between thermal therapy and intravesical chemotherapy after subgroup analysis stratified by chemotherapeutic agent used in thermal intravesical chemotherapy.

All studies were available, including 888 patients, 445 in the thermal intravesical chemotherapy group and 443 in the normal temperature intravesical chemotherapy group. The meta-analysis demonstrated a significant difference in recurrence rate between thermal intravesical chemotherapy with normal temperature intravesical chemotherapy in different follow-up groups (24 months follow-up group: RR = 0.30, 95% CI: 0.21–0.43, *P* < 0.00001, *I*^2^ = 0%; 36 months follow-up group: RR = 0.27, 95% CI: 0.14–0.54, *P* = 0.0002, *I*^2^ = 0%; Figure 3). The publishing bias are limited (*P* = 0.95, *I*^2^ = 0%; Figure 4). Subgroup analysis shows that both HIVEC and ETFT vs. normal temperature intravesical chemotherapy confirm a significant difference statistically (RR = 0.34, 95% CI: 0.25–0.46, *P* < 0.00001 and RR = 0.31, 95% CI: 0.20–0.50, *P* < 0.00001; Figure 5). As for the different drugs used in the thermal intravesical chemotherapy, MMC and THP both can reduce the recurrence rate (RR = 0.33, 95% CI: 0.24–0.46, *P* < 0.00001 and RR = 0.30, 95% CI: 0.18–0.51, *P* < 0.00001; Figure 6).

### Adverse Event Rate

**Figure 7:**
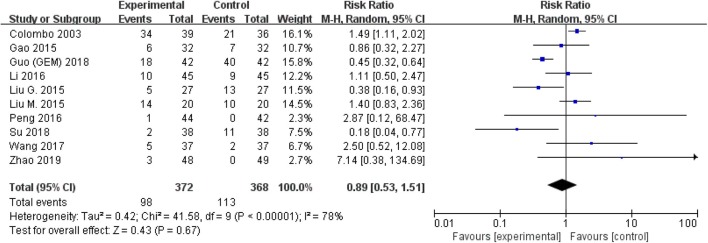
Forest plot of adverse event rate comparing thermal intravesical chemotherapy with normal temperature intravesical chemotherapy.

**Figure 8:**
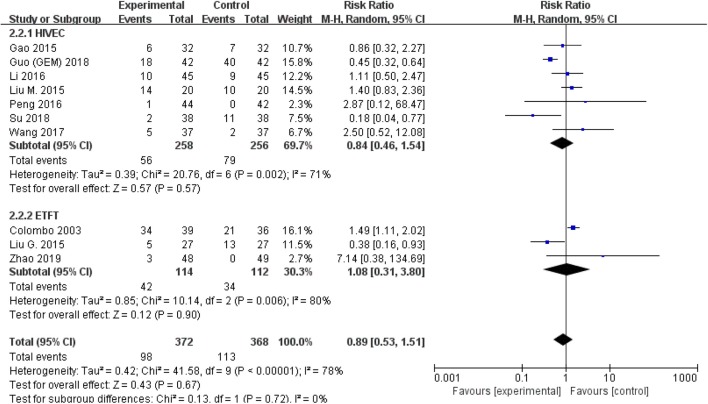
Comparison on adverse event rate between thermal intravesical chemotherapy and normal temperature intravesical chemotherapy after subgroup analysis stratified by different approach used in thermal therapy.

**Figure 9:**
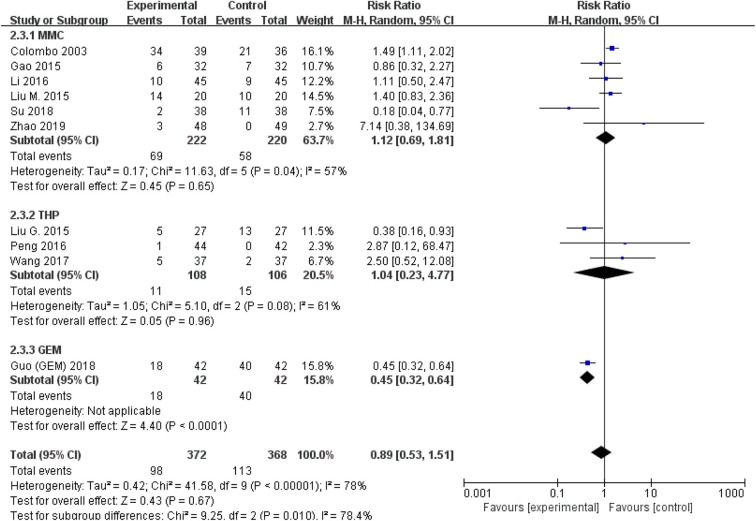
Comparison on adverse event rate between thermal intravesical chemotherapy and normal temperature intravesical chemotherapy after subgroup analysis stratified by chemotherapeutic agent used in thermal intravesical chemotherapy.

The comparison of adverse events rate involved 10 studies (*n* = 740) because the other two studies were double-counted. Thermal intravesical chemotherapy seemed no more toxic than normal temperature intravesical chemotherapy (RR = 0.89, 95% CI: 0.53–1.51, *P* = 0.67; Figure 7). Subgroup analysis shows that the adverse events rate of thermal intravesical chemotherapy using different methods (HIVEC group: RR = 0.84, 95% CI: 0.46–1.54, *P* = 0.57; ETFT group: RR = 1.08, 95% CI: 0.31–3.80, *P* = 0.90; Figure 8) or different drugs (MMC group: RR = 1.12, 95% CI: 0.69–1.81, *P* = 0.65; THP group: RR = 1.04, 95% CI: 0.23–4.77, *P* = 0.96; Figure 9) was not statistically different from that at normal temperature.

## Discussion

The idea that thermal therapy can treat the tumor can be traced back to 1910 from Coley (27). And Rigatti et al. first applied thermal therapy to treat superficial bladder tumors in 1991 (28). Then Colombo et al. used a microwave device to make local bladder heating for intravesical chemotherapy, with a good overall response rate of 90.8% in 44 superficial bladder cancer patients (29). As the encouraging results were achieved, more and more urologists join to investigate thermal intravesical chemotherapy. Van der Heijden et al. reported 90 patients received combined treatment of MMC and local microwave hyperthermia. Finally, the risk of recurrence was 24.6% after a 2 year follow-up and no one suffered from stage or grade progression (30). Fifty-two patients with high-grade NMIBC treated by Gofrit et al. had chemo-thermotherapy, 86.5% of these patients preserved their bladder in the end of s 23 month follow-up (31). All these literature show the same point that thermal intravesical chemotherapy demonstrated a tumor cell killing effect which might be a good for NMIBC patients. The meta-analysis aimed to figure out whether thermal intravesical chemotherapy is more effective and safer than normal temperature intravesical chemotherapy.

In the present study, the results of 12 eligible studies, include 11 RCTs and 1 retrospective study, were analyzed. Although the adverse events rate was not reduced, it highlighted that thermal intravesical chemotherapy was more advantageous than normal temperature intravesical chemotherapy in reducing risk of tumor recurrence among patients with NMIBC. Subgroup analyses of different chemotherapy approaches and drugs also indicated a significant reduction in recurrence rate without increasing adverse event rate.

The meta-analysis shows that the thermal intravesical chemotherapy can be tolerated relatively by patients. Most treatment adverse events were localized and transient as reporting. Of all the intake studies, only Zhao et al. (15, 25) and Colombo et al. (16) reported 12 and 8 patients' discontinuation, respectively. Reasons for withdrawal were various but mostly drug allergy, while Geijsen et al. (10) reported the oldest patients withdrew from complaints of improper hyperthermia treatment positioning. Our meta-analysis shows a lower withdrawal rate of 1.4%, which is close to 3.8% of Lammers's research (14).

There are two approaches to execute thermal intravesical chemotherapy. The first kind is called hyperthermic intravesical chemotherapy. In this way, chemotherapeutic agents dissolve in the solution and put it into a hyperthermic perfusion treatment machine connect with pipeline systems for infusion. The advantage of this method is obvious that we can control the temperature constantly. But still we cannot detect the temperature in the bladder wall since it became thinner during perfusion (27). Moreover, in consideration of single-use pipes, it may be too expensive to afford, especially for NMIBC patients who should generally receive more than six rounds intravesical chemotherapy. The second choice, as we called external thermal field thermotherapy, creates the local bladder heating by microwave. This method is more convenient and cheaper without pipeline systems (15). The weakness of ETFT is the intravesical temperature which could not be supervised with high-precision.

As for chemotherapeutic agents, there are four kinds of drugs used in thermal intravesical chemotherapy in our meta-analysis, including mitomycin C, pirarubicin, gemcitabine, hydroxycamptothecine. Only Zhao et al. compared the efficacy between thermal intravesical chemotherapy and normal temperature BCG perfusion (15), and the result turned out to be no difference in recurrence rate after the 2 years follow-up. BCG has been proved to be the most effective treatment for intermediate- and high-risk NMIBC since its first report in 1976 and became the standard management. Despite its efficacy, BCG perfusion therapy could cause a variety of adverse events, leading to the termination of the treatment in the end (7). More badly, BCG is scarce and expensive in China (15). So we have an urgent need to develop alternative therapy. MMC and THP are the main drugs applied in studies. MMC is an anti-metabolite drug identified from the products of a species of *Streptomyces caespitosus* (32). It destroys the structure and function of DNA, inhibits the replication of DNA, and kills tumor cells both in the proliferating and resting phase. The advantage of MMC is that normal mucosa of bladder is resistant to it. So patients who received MMC intravesical chemotherapy have fewer adverse events (33). THP can be absorbed into tumor cells quickly as it is one of the most effective agents for reducing recurrence of NMIBC (34). Although it shows no difference in the present meta-analysis, the study of the efficacy of the different thermal intravesical chemotherapeutics should be explored further.

As we all know, before thermal intravesical chemotherapy arises, intraperitoneal perfusion chemotherapy has already been widely used in the treatment of advanced ovarian and gastric cancer or peritoneal metastases. Zhu et al. reported patients after D2 dissection received intraperitoneal chemotherapy with whole abdominal hyperthermia, which reduced the recurrence and metastasis of peritoneal (35). Hotouras et al. systematically reviewed the literature of heated intraperitoneal chemotherapy (HIPEC) for recurrent ovarian cancer patients, and found that HIPEC is associated with benefits (36). Foreseeable, larger prospective studies will be carried out in all these fields.

In China, according to epidemiological investigation report published in 2018, bladder cancer has the highest morbidity among all kinds of urogenital neoplasms. The morbidity of male and female are 8.65 and 2.62 per 100,000 (37). The situation demands immediate action, especially treatment for NMIBC. In this meta-analysis, we found that thermal intravesical chemotherapy can reduce the recurrence rate of NMIBC. It indicates that thermal intravesical chemotherapy might be the next hot topic in academia.

## Limitations

There are some limitations remaining, in spite of the eligibility criteria, that we had set to select published literature. First of all, treatment schedules and chemotherapeutic agents used in thermal intravesical chemotherapy are heterogeneous indeed. Second, risk levels among patients varied, which make it impossible to evaluate the difference in efficacy between thermal intravesical chemotherapy and normal temperature intravesical chemotherapy for patients at specific risk levels. These may bias the conclusions of this study. Beyond these limitations, this meta-analysis was strictly performed with setting reasonable eligibility criteria and reviewing all available publications' data, thus comparing efficacy between thermal intravesical chemotherapy and normal temperature intravesical chemotherapy.

## Conclusions

In summary, compared with intravesical chemotherapy, thermal intravesical chemotherapy was associated with a lower recurrence rate without increasing adverse event rate among patients with NMIBC. Different approaches and drugs show the same effects of the efficacy. More high quality RCTs are still required to confirm those conclusions.

## Data Availability Statement

All datasets generated for this study are included in the article/Supplementary Material.

## Author Contributions

This meta-analysis was designed by X-QL and JZ. Searching of literature and data extraction was performed by KL and K-CZ. Data was rechecked by Y-XS and YL. Statistical analysis was performed by KL and XW. Writing of the manuscript was performed by KL and JZ. KL polished the article's English. X-QL reviewed the manuscript. All authors read and approved the final manuscript.

### Conflict of Interest

The authors declare that the research was conducted in the absence of any commercial or financial relationships that could be construed as a potential conflict of interest.

## References

[1] Dobruch J Daneshmand S Fisch M Lotan Y Noon AP Resnick MJ. Gender and bladder cancer: a collaborative review of etiology, biology, and outcomes. Euro Urol. (2016) 69:300–10. 10.1016/j.eururo.2015.08.037 26346676

[2] Bray F Ferlay J Soerjomataram I Siegel RL Torre LA Jemal A. Global cancer statistics 2018: GLOBOCAN estimates of incidence and mortality worldwide for 36 cancers in 185 countries. Cancer J Clin. (2018) 68:394–424. 10.3322/caac.21492 30207593

[3] Huang J Liu H Current status of management of non-muscle invasive bladder cancer. Chin J Urol. (2019) 40:481–4.

[4] Woldu SL Bagrodia A Lotan Y. Guideline of guidelines: non-muscle-invasive bladder cancer. BJU Int. (2017) 119:371–80. 10.1111/bju.13760 28058776 PMC5315602

[5] Kamat AM Colombel M Sundi D Lamm D Boehle A Brausi M. BCG-unresponsive non-muscle-invasive bladder cancer: recommendations from the IBCG. Nat Rev Urol. (2017) 14:244–55. 10.1038/nrurol.2017.16 28248951

[6] Kamat AM Bellmunt J Galsky MD Konety BR Lamm DL Langham D Society for immunotherapy of cancer consensus statement on immunotherapy for the treatment of bladder carcinoma. J Immunother Cancer. (2017) 5:68 10.1186/s40425-017-0271-0 28807024 PMC5557323

[7] Kawai K Miyazaki J Joraku A Nishiyama H Akaza H. Bacillus Calmette-Guerin (BCG) immunotherapy for bladder cancer: current understanding and perspectives on engineered BCG vaccine. Cancer Sci. (2013) 104:22–7. 10.1111/cas.12075 23181987 PMC7657210

[8] Joice GA Bivalacqua TJ Kates M. Optimizing pharmacokinetics of intravesical chemotherapy for bladder cancer. Nat Rev Urol. (2019) 16:599–612. 10.1038/s41585-019-0220-4 31434998

[9] Abern MR Owusu RA Anderson MR Rampersaud EN Inman BA. Perioperative intravesical chemotherapy in non-muscle-invasive bladder cancer: a systematic review and meta-analysis. J Natl Compr Cancer Netw. (2013) 11:477–84. 10.6004/jnccn.2013.0060 23584348

[10] Geijsen ED de Reijke TM Koning CC Zum Vorde Sive Vording PJ de la Rosette JJ Rasch CR. Combining mitomycin C and regional 70 MHz hyperthermia in patients with nonmuscle invasive bladder cancer: a pilot study. J Urol. (2015) 194:1202–8. 10.1016/j.juro.2015.05.102 26143111

[11] Siddiqui J Brown K Zahid A Young CJ. Current practices and barriers to referral for cytoreductive surgery and HIPEC among colorectal surgeons: a binational survey. Euro J Surg Oncol. (2019) 46: 166–72. 10.1016/j.ejso.2019.09.007 31542240

[12] Ekin RG Akarken I Cakmak O Tarhan H Celik O Ilbey YO. Results of intravesical chemo-hyperthermia in high-risk non-muscle invasive bladder cancer. Asian Pac J Cancer Prev. (2015) 16:3241–5. 10.7314/APJCP.2015.16.8.3241 25921126

[13] Tan WS Kelly JD. Intravesical device-assisted therapies for non-muscle-invasive bladder cancer. Nat Rev Urol. (2018) 15:667–85. 10.1038/s41585-018-0092-z 30254383

[14] Lammers RJ Witjes JA Inman BA Leibovitch I Laufer M Nativ O. The role of a combined regimen with intravesical chemotherapy and hyperthermia in the management of non-muscle-invasive bladder cancer: a systematic review. Euro Urol. (2011) 60:81–93. 10.1016/j.eururo.2011.04.023 21531502

[15] Zhao Z Zhao G Zheng D Chen S Li J Lin S Clinical analysis of adjuvant hyperthermic intravesical chemotherapy for treating non-muscle invasive bladder cancer. Int J Urol Nephrol. (2019) 39:450–3.

[16] Colombo R Da Pozzo LF Salonia A Rigatti P Leib Z Baniel J. Multicentric study comparing intravesical chemotherapy alone and with local microwave hyperthermia for prophylaxis of recurrence of superficial transitional cell carcinoma. J Clin Oncol. (2003) 21:4270–6. 10.1200/JCO.2003.01.089 14581436

[17] Gao F Wang G Intravesical mitomycin hyperthemal perfusion combined withItransurethral resection for treating bladder cancer in 32 cases. Chin Pharm. (2015) 24:85–6.

[18] Guo X Wang M Shi L Bai Y Clinical observation of immediate intravesical instillation of gemcitabine hyperthermic perfusion and normal temperature perfusion after TURBt in elderly patients with high risk non-muscle invasive bladder cancer. J Clin Urol. (2018) 33:821–4.

[19] Guo X Wang M Shi L Bai Y Clinical observation of immediately hyperthermic intravesical chemotherapy with pirarubicin versus traditional intravesical instillation with pirarubicin after transurethral resection of the bladder tumor in elderly patients with superficial bladder cancer. Pract Geriatr. (2018) 32:524–6.

[20] Li B Xia T Effect of BR-TRG-I body cavity hyperthermic perfusion instrument on intravesical chemotherapy. J Clin Urol. (2016) 31:419–21.

[21] Liu G Teng D Huang G Cao J Sun W Clinical application of thermochemotherapy for bladder cancer. Med Inf. (2015) 28:87.

[22] Liu M Zhang Y Curative effect comparative analysis of hyperthermic perfusion therapy and mitomycin perfusion chemotherapy after transurethral resection of bladder tumor. Chin Foreign Med Res. (2015) 13:17–8.

[23] Peng Y Feng Z Huang Q Qiu G Efficacy of pirobicin in the prevention of recurrence of superficial bladder cancer after TURBT. China Prac Med. 2016;11(1):176–8.

[24] Su X Pan X Liu Y Observation on the efficacy of mitomycin hyperthermic intravesical chemotherapy combined with TURBT in patients with non-muscle invasive bladder cancer. Chin J Mod Drug Appl. (2018) 12:105–6.

[25] Zhao Z Hu W Zheng D Zhao G Chen S Chen H Preventing recurrence of superficial bladder cancer with intravesical chemotherapy of HCPT combined with external electric field hyperthermia. J Mod Urol. (2014) 19:508–12.

[26] Wang Y Li Y Hong J Li W Zheng S Wu K The primary clinical application of hyperthermic intravesical chemotherapy in the prevention of non-muscle-invasive bladder cancer recurrence after transurethral resection of bladder tumor. J Mod Urol. (2017) 22:903–6.

[27] Coley WB. The treatment of inoperable sarcoma by bacterial toxins (the mixed toxins of the *Streptococcus erysipelas* and the *Bacillus prodigiosus*). Proc R Soc Med. (1910) 3:1–48. 10.1177/003591571000301601 19974799 PMC1961042

[28] Rigatti P Lev A Colombo R. Combined intravesical chemotherapy with mitomycin C and local bladder microwave-induced hyperthermia as a preoperative therapy for superficial bladder tumors. A preliminary clinical study. Euro Urol. (1991) 20:204–10. 10.1159/000471701 1823044

[29] Colombo R Lev A Da Pozzo LF Freschi M Gallus G Rigatti P. A new approach using local combined microwave hyperthermia and chemotherapy in superficial transitional bladder carcinoma treatment. J Urol. (1995) 153(3 Pt 2):959–63. 10.1016/S0022-5347(01)67613-4 7853583

[30] van der Heijden AG Kiemeney LA Gofrit ON Nativ O Sidi A Leib Z Preliminary European results of local microwave hyperthermia and chemotherapy treatment in intermediate or high risk superficial transitional cell carcinoma of the bladder. Euro Urol. (2004) 46:65–71. 10.1016/j.eururo.2004.01.019 15183549

[31] Gofrit ON Shapiro A Pode D Sidi A Nativ O Leib Z. Combined local bladder hyperthermia and intravesical chemotherapy for the treatment of high-grade superficial bladder cancer. Urology. (2004) 63:466–71. 10.1016/j.urology.2003.10.036 15028439

[32] Serretta V Scalici Gesolfo C Alonge V Di Maida F Caruana G. Mitomycin C from birth to adulthood. Urologia. (2016) 83(Suppl 2):2–6. 10.5301/uro.5000195 27716885

[33] Bosschieter J Nieuwenhuijzen JA van Ginkel T Vis AN Witte B Newling D. Value of an immediate intravesical instillation of mitomycin C in patients with non-muscle-invasive bladder cancer: a prospective multicentre randomised study in 2243 patients. Euro Urol. (2018) 73:226–32. 10.1016/j.eururo.2017.06.038 28705539

[34] Kang M Jeong CW Kwak C Kim HH Ku JH. Single, immediate postoperative instillation of chemotherapy in non-muscle invasive bladder cancer: a systematic review and network meta-analysis of randomized clinical trials using different drugs. Oncotarget. (2016) 7:45479–88. 10.18632/oncotarget.9991 27323781 PMC5216735

[35] Zhu L Xu Y Shan Y Zheng R Wu Z Ma S. Intraperitoneal perfusion chemotherapy and whole abdominal hyperthermia using external radiofrequency following radical D2 resection for treatment of advanced gastric cancer. Int J Hyperther. (2019) 36:403–7. 10.1080/02656736.2019.1579372 30829551

[36] Hotouras A Desai D Bhan C Murphy J Lampe B Sugarbaker PH. Heated IntraPEritoneal Chemotherapy (HIPEC) for patients with recurrent ovarian cancer: a systematic literature review. Int J Gynecol Cancer. (2016) 26:661–70. 10.1097/IGC.0000000000000664 26844612

[37] He Y Li D Liang D Zheng R Zhang S Zeng H. Incidence and mortality of bladder cancer in China, 2014. Chin J Oncol. (2018) 40:647–52. 10.3760/cma.j.issn.0253-3766.2018.09.002 30293387

