# Supplementary material for: Thermal Intravesical Chemotherapy Reduce Recurrence Rate for Non-muscle Invasive Bladder Cancer Patients: A Meta-Analysis
Source: Front Oncol. 2020 Feb 5;10:29. doi: 10.3389/fonc.2020.00029 (PMC7015071; doi:10.3389/fonc.2020.00029)
Supplement: Supplementary file 1 [file Data_Sheet_1.docx]

**Appendix I**

#1 Transitional cell carcinoma urinary bladder

#2 Urothelial cell carcinoma urinary bladder

#3 Urinary bladder cancer

#4 Urinary bladder neoplasm

#5 Urinary bladder tumor

#6 Urinary bladder tumour

#7 #1 OR #2 OR #3 OR #4 OR #5 OR #6

#8 Papill*

#9 Superficial

#10 Non-muscle invasive

#11 Non-invasive

#12 Meta*

#13 Advanced

#14 #8 OR #9 OR #10 OR #11 NOT #12 NOT #13

#15 #7 AND #14

#16 Thermo-chemotherapy

#17 Thermo chemotherapy

#18 Thermal therapy

#19 Thermal intravesical chemotherapy

#20 Microwave induced local hyperthermia

#21 External thermal field thermotherapy

#22 Hyperthermia

#23 Hyperthermic intravesical chemotherapy

#24 #16 OR #17 OR #18 OR #19 OR #20 OR #21 OR #22 OR #23

#25 #15 AND #24
